# Neurological manifestations of immune origin after COVID-19 vaccination: retrospective case study

**DOI:** 10.3389/fphar.2024.1376474

**Published:** 2024-08-08

**Authors:** Juan Granja López, Carlos Estebas Armas, Manuel Lorenzo Dieguez, Inmaculada Puertas Muñoz, Elena De Celis Ruiz, Ricardo Rigual, Mireya Fernández-Fournier, Gabriel Torres Iglesias, Sara Sánchez Velasco, Antonio Tallón Barranco, Olga Rogozina, Elena Ramírez, Miguel González-Muñoz, Laura Lacruz Ballester

**Affiliations:** ^1^ Neurology Department and Stroke Center, Laboratory of Neurological and Cerebrovascular Sciences, Hospital La Paz Institute for Health Research Institute-IdiPAZ (La Paz University Hospital—Universidad Autónoma de Madrid), Madrid, Spain; ^2^ Clinical Pharmacology Department, La Paz University Hospital-IdiPAZ, Faculty of Medicine, Universidad Autónoma de Madrid, Madrid, Spain; ^3^ Immunology Department, La Paz University Hospital-IdiPAZ, Madrid, Spain

**Keywords:** COVID-19 vaccination, autoimmune disorders, polyradiculoneuropathy, cranial neuropathy, Spanish pharmacovigilance system causality algorithm, lymphocyte transformation test

## Abstract

**Objectives:** To know the frequency and characteristics of neurological manifestations of probable immune origin occurring after exposure to COVID-19 vaccination. In addition, to pre-study the usefulness of the Spanish pharmacovigilance system and lymphocyte transformation test in establishing causality.

**Methods:** Retrospective case study, including patients admitted to the Neurology department from January 2021 to May 2022 with a probable neuroimmune disorder. Demographic, clinical and COVID-19 vaccination antecedent data were collected from medical records.

**Results:** From a total of 108 patients, 30 were excluded due to a different etiological diagnosis after follow-up. Thirty-six patients (46.2%) had received the COVID-19 vaccine in the previous 3 months (21.8% during the previous month). BioNTech-Pfizer vaccine was the most frequent in this group (63.9%). 69/108 were female and mean age 51.2 years (SD 22.59), with no significant difference with not recently-vaccinated (U-Mann Whitney, *p* = 0.256). The neurological syndromes found were (vaccinated/total): polyradiculoneuropathy (8/16), encephalitis (5/11), multiple sclerosis relapse (5/16), optic neuritis (1/4), myelitis (3/6), cranial neuropathy (6/10), aseptic meningitis (1/3) and others (7/11). Acute immunosuppressive treatment was administered in 61.1% of cases and 47.2% presented complete clinical improvement, without significant differences with non-vaccinated patients (chi-square, *p* = 0.570). Eleven vaccinated patients were studied in the pharmacovigilance office for possible adverse drug reaction. Causality according to the Spanish pharmacovigilance system (SPVS) algorithm was “Related” to COVID-19 vaccine (score ≥ 4) in 11 cases with positive *in vitro* study (lymphocyte transformation test) to polyethylene glycol-2000 and polysorbate-80 in 4 cases.

**Conclusion:** Neuroimmune disorders appearing after administration of COVID-19 vaccine do not seem to present important differentiating clinical and/or evolutive features. Delayed hypersensitivity to vaccine excipients could be one of the pathophysiological mechanisms, and lymphocyte transformation test is a useful tool to identify it.

## 1 Introduction

COVID-19 has been a pandemic that has affected, up to the time of the writing of this article, more than 760 million people worldwide. Thus, the production of vaccines was enormously accelerated, leading to the development of effective COVID-19 vaccines in a relatively short period of time. Four main types of vaccines against SARS-Cov-2: nucleic acid (mRNA or DNA), spike protein-based, viral vector and whole attenuated or inactivated virus have been used.

All vaccines have demonstrated to be effective tools reducing the number of severe COVID-19 cases, hospitalization, and death (Fiolet et al., 2022). On the other hand, multiple related adverse effects have been reported, the most common ones being pain and swelling at the site of the injection, fever, myalgias and fatigue (Al Khames Aga et al., 2021). Besides these local and systemic symptoms, several neurologic adverse events were also reported following SARS-CoV2 vaccines. Most of them are mild, brief and can be managed on an outpatient basis. However, there are cases of more severe neurological disorders, requiring admission to hospital. The most devastating post-vaccination neurological complication is cerebral venous sinus thrombosis, generally following adenovector-based vaccination (García-Azorín et al., 2022). Bell’s palsy and herpes zoster reactivation have also been reported in some people after administration of mRNA vaccines. Other serious neurological complications include acute transverse myelitis, acute disseminated encephalomyelitis, and acute demyelinating polyneuropathy (Walker et al., 2022; Chatterjee and Chakravarty, 2023).

The relationship of these neuroimmune syndromes with the vaccine is disputed, as it is complicated to demonstrate a cause-effect relationship. Bradford Hill defined 9 criteria needed to establish such a relationship: 1) strength of association, 2) consistency, 3) specificity of the effect, 4) temporal association, 5) biological gradient, 6) biological plausibility, 7) coherence, 8) experimentation and 9) reasoning by analogy. In pharmacovigilance, these criteria are carried out by means of causality algorithms, one of these being the Spanish pharmacovigilance system (SPVS) algorithm, a modified version of Karch-Lasagna algorithm. These algorithms have high (nearly 100%) sensitivity and positive predictive value, but low (not higher than 37.5%) specificity and negative predictive values (Macedo et al., 2006). More accurate tests to confirm a physiopathological association between vaccines and neurological syndrome are necessary.

The four most frequently administered SARS-Cov2 vaccines in Spain are *Comirnaty* by *Pfizer/BioNTech*, *Spikevax* by *Moderna/Lonza*, *Vaxzevria* by *Oxford/AstraZeneca* and *Jcovden* by *Janssen/Cilag*. The mechanisms of action were through mRNA in the first two, and viral vectors in the latter two. A number of these vaccines contain new ingredients that had not previously been employed in vaccine manufacturing. The mRNA encoding the spike protein is encapsulated in lipid nanoparticles containing lipids and polyethylene glycol (PEG) 2000, which differs from the PEG used in other vaccines and healthcare products and serves as a stabilizer to prevent rapid enzymatic degradation of mRNA and facilitate *in vivo* delivery in the *Pfizer-BioNTech* and *Moderna* vaccines. *Moderna*’s vaccine also contains trometamol as an excipient, whereas *AstraZeneca*’s and *Johnson and Johnson*’s vaccines contain polysorbate 80 (P80), a nonionic surfactant and emulsifier often used in foods and cosmetics (Cabanillas and Novak, 2021; Lamprinou et al., 2023).

Among all the laboratory techniques used for the study of adverse reactions to drugs, lymphocyte transformation test (LTT) is based on the detection of drug-specific proliferating memory T cells, whose production is a common starting point for the immune response (Sachs et al., 2021). Preliminary results indicate that LTT with PEGs and polysorbates is a useful tool for identifying excipients as causal agents in delayed adverse reactions to COVID-19 vaccines (Ruiz-Fernández et al., 2023) and other events not related to vaccines (Rogozina et al., 2024).

This retrospective study aims to identify the specific characteristics of post-vaccination neurological disorders and assess the effectiveness of a clinical protocol in determining the degree of causality (using the SPVS algorithm) and potential distinctive immunological mechanisms (such as hypersensitivity reactions to excipients, using the LTT). The goal is to identify the neurological manifestations occurring after receiving the COVID-19 vaccine and make a risk evaluation to determine the most suitable type of revaccination in these patients.

## 2 Materials and methods

### 2.1 Patients

Adult patients with neurological syndromes of probable immune-mediated etiology, admitted to Neurology at Hospital Universitario La Paz, from January 2021 to May 2022, were included in this case study. Demographic, clinical, and COVID-19 vaccination data were retrospectively collected from the electronic medical record, accessed through the Health Care Information System (HCIS).

If a non-immune-mediated etiology, or other possible triggers like previous infectious or neoplasia, were identified during admission, the case was excluded. Relapses of chronic autoimmune neurological diseases (e.g., multiple sclerosis) were also included. The time limit established as recent vaccination was 90 days, since this is the proposed pharmacovigilance period of time for vaccines (Butler et al., 2021; Case definitions, 2023).

In addition, the Clinical Pharmacology Department was asked to evaluate those patients that, following a comprehensive neurological examination, were suspected to have a condition potentially related to the vaccine. A causality assessment using the SPVS algorithm and a lymphocyte transformation test for the vaccine excipients polyethylene glycol 2000 and polysorbate 80 were conducted in these cases.

This study was approved by the La Paz University Hospital Ethics Committee. Due to study’s retrospective nature, no intervention and data anonymization, the absence of informed consent was allowed.

### 2.2 Causality assessment

The causality assessment was performed using the Spanish pharmacovigilance system (SPVS) algorithm. This algorithm is a modification of the Karch and Lasagna algorithm (Karch and Lasagna, 1977). It consists of seven criteria: 1) time sequence, compatibility of the time between the drug or vaccine and the adverse effect, taking into account the pathophysiological process taking place; 2) prior knowledge of the adverse effect in the literature; 3) evolution of the adverse effect after withdrawal; 4) effect of re-exposure to the suspected substance; 5) existence of alternative causes; 6) contributing factors favoring a causal relationship; and 7) results of complementary explorations (drug levels, biopsy, imaging, etc.). Each of the first five criteria have different degrees of association (from 4 to 8), to each of which corresponds a score (from −3 to +3, a higher score with a stronger association). The last two criteria only have two answers with a score of +1 or 0. The causal relationship is then classified into five categories depending on the final score: < 0, unrelated; 1-3, conditional; 4–5, possible; 6–7, probable; and 8, definite. The designation of “possible” in causality assessment represents the minimum threshold for a case to be considered drug-related (score ≥ 4) (Aguirre and García, 2016).

### 2.3 Lymphocyte transformation test

In the LTT, peripheral blood mononuclear cells (PBMC) of the patient are co-incubated with the ingredient of the vaccine. The patient’s antigen-presenting cells can then present the antigen to the drug-specific memory T cells, leading to their activation and expansion. The 3H-thymidine is incorporated in the DNA of the new cells, enabling quantification of the proliferation by measuring the radiation.

LTT was performed using several concentrations of the excipients PEG 2000 (CAS 25322-68-3) and P80 (CAS 9005-65-6; *ThermoFisher Scientific, Madrid, Spain*). LTT was performed after event recovery and at least 1 month after steroid therapy was stopped, if applicable. Lymphocyte proliferation was measured as previously described (Pichler and Tilch, 2004). Mononuclear cells were separated over a density gradient (Histopaque–1077, *Sigma-Aldrich, Madrid, Spain*) from fresh peripheral blood and were plated in flat bottom wells of microtiter plates at 2 × 10^5^ cells/well. Cells were incubated for 6 days with various excipient concentrations (10 µg/mL–0.01 μg/mL) in triplicate. We used phytohemagglutinin (PHA, 5 μg/mL) as a positive control. The excipient concentration curve was previously assayed for toxicity, adding the excipients to PHA-stimulated cell cultures from three controls. For the final 18 h of the incubation period, proliferation was determined by adding 1 µCi [3^H^] of thymidine. Proliferative responses were calculated as the stimulation index (SI), defined as the ratio between the mean values of the counts per minute in cultures with the drug and those obtained without the drug.

### 2.4 Statistical analysis

Comparative analysis of variables between recently and not recently vaccinated patients was performed using IBM SPSS Statistics version 21.0 (IBM Corporation, Armonk, NY, United States). Continuous variables are expressed as mean and standard deviation (SD) or median and range according to the Kolmogorov–Smirnov normality test. Categorical variables are expressed in absolute terms and percentages. Student’s T or Mann Whitney’s U were used to analyze continuous variables, as appropriate. The chi square was used to analyze the categorical variables.

## 3 Results

### 3.1 Included patients

**FIGURE 1:**
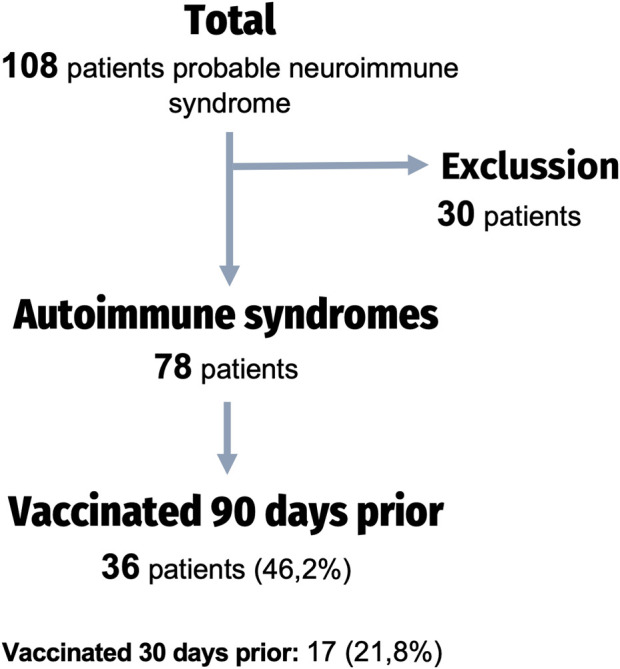
Flow chart of the study.

**FIGURE 2:**
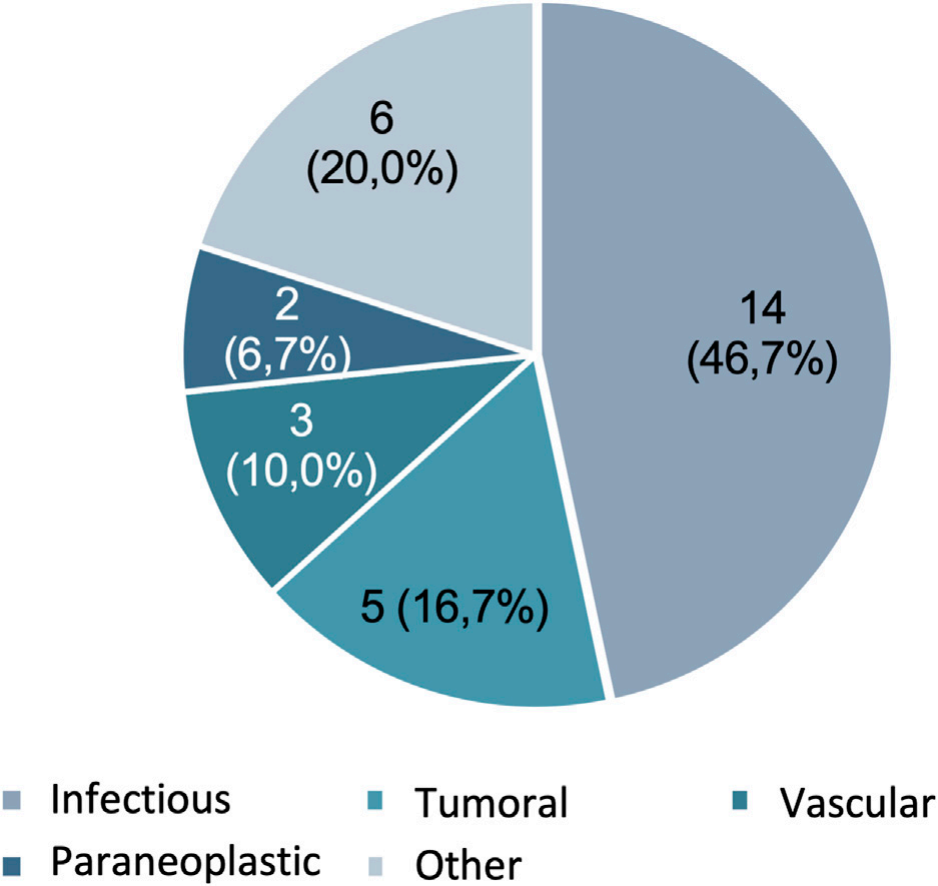
Etiology of the neurological syndrome of the excluded cases: n (%).

108 patients were admitted to our hospital with an initial suspicion of a neuroimmune disorder. Following a comprehensive study, 30 of these patients were ultimately excluded for not having an immunization-mediated etiology (Figure 1). The most frequent etiology of exclusion was infectious (n = 14 patients, 46.7%), followed by tumoral (n = 5 patients, 41.7%) (Figure 2).

### 3.2 Baseline characteristics

The characteristics of our sample are summarized in Table 1. Thirty-six patients (46.2%) were identified in temporal relation to the SARS-CoV-2 vaccine (previous 90 days). The remaining 42 were either not vaccinated (n = 15; 19.2%) or had been vaccinated more than 90 days prior to the onset of the neurological symptoms (n = 27; 34.6%). The mean age of the total sample was 48.5 years (SD 21.7) and 53.8% were women, with no significant differences between those recently vaccinated (V90) and those non recently vaccinated (NV90).

**TABLE 1:** Baseline characteristics of vaccinated and non-vaccinated patients in the 90 days prior to the onset of the neurological symptoms.

	Total n = 78	Vaccinated 90 days prior n = 36	Not vaccinated 90 days prior n = 42	*p*-value
Age Average years (SD)	48.5 (21.7)	51.19 (22.59)	48.1 (21.2)	0.26^a^
Female n (%)	42 (53.8)	23 (63.9)	19 (45.0)	0.10^a^
Prior COVID-19 n (%)	17 (21.8)	8 (22.2)	9 (21.4)	0.65^b^
Prior autoimmune diseases n (%)	7 (9.0)	3 (8.3)	4 (9.5)	

^a^ Mann Whitney’s U.

^b^ Chi square.

21.8% of total cases had suffered from a previous SARS-CoV-2 infection (22.2% in V90 group and 21.4% in NV90 group). The median number of days between SARS-CoV-2 infection and the onset of symptoms of neuroimmune disorder was 119 days in the V90 group and 12.5 in NV90 group (Mann-Whitney U; *p* = 0.014).

Seven patients had a history of previous systemic autoimmune disease (9%), three patients in V90 group (8.3%) and four patients in the NV90 group (9.5%).

### 3.3 SARS-COV2 vaccine

The majority of recently vaccinated patients received the *BioNTech/Pfizer* vaccine (63.9%). This was followed in frequency by the *Moderna/Lonza* vaccine (19.4%) and *Oxford/AstraZeneca* (13.9%).

**FIGURE 3:**
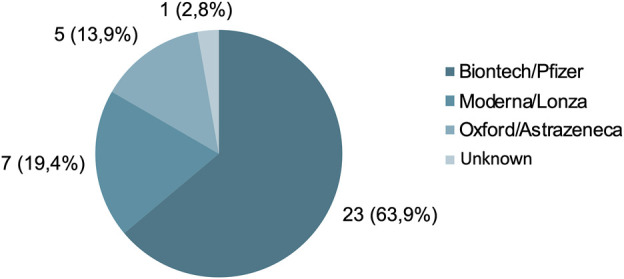
Type of vaccine received: n (%).

In addition, it was most frequent to have received two doses of vaccination (n = 16; 44.4%), although closely followed by those who had received a single dose (n = 15; 41.6%). Only five patients had received three doses (13.9%) (Figure 3).

### 3.4 Immune-mediated neurological disorders

Different types of dysimmune neurological syndromes were found: a) Polyradiculoneuropathies (total n = 16; V90 n = 8; NV90 n = 8); b) Multiple sclerosis relapses (total n = 16; V90 n = 5; NV90 n = 11), c) Encephalitis (total n = 11; V90 n = 5; NV90 n = 6); d) Cranial neuropathies (total n = 10; V90 n = 6; NV90 n = 4); e) Myelitis (other than multiple sclerosis) (total n = 6; V90 n = 3; NV90 n = 3); f) Optic neuritis (total n = 4; V90 n = 1; NV90 n = 3); g) Aseptic meningitis (total n = 3; V90 n = 1; NV90 n = 2); and h) Others: sensorimotor syndrome of lower extremities without criteria for a polyradiculoneuropathy; aphasic syndrome without abnormalities on image; new onset focal epilepsy without brain lesions; cryptogenic vasculitis or Susac syndrome (total n = 11; V90 n = 7; NV90 n = 4).

**FIGURE 4:**
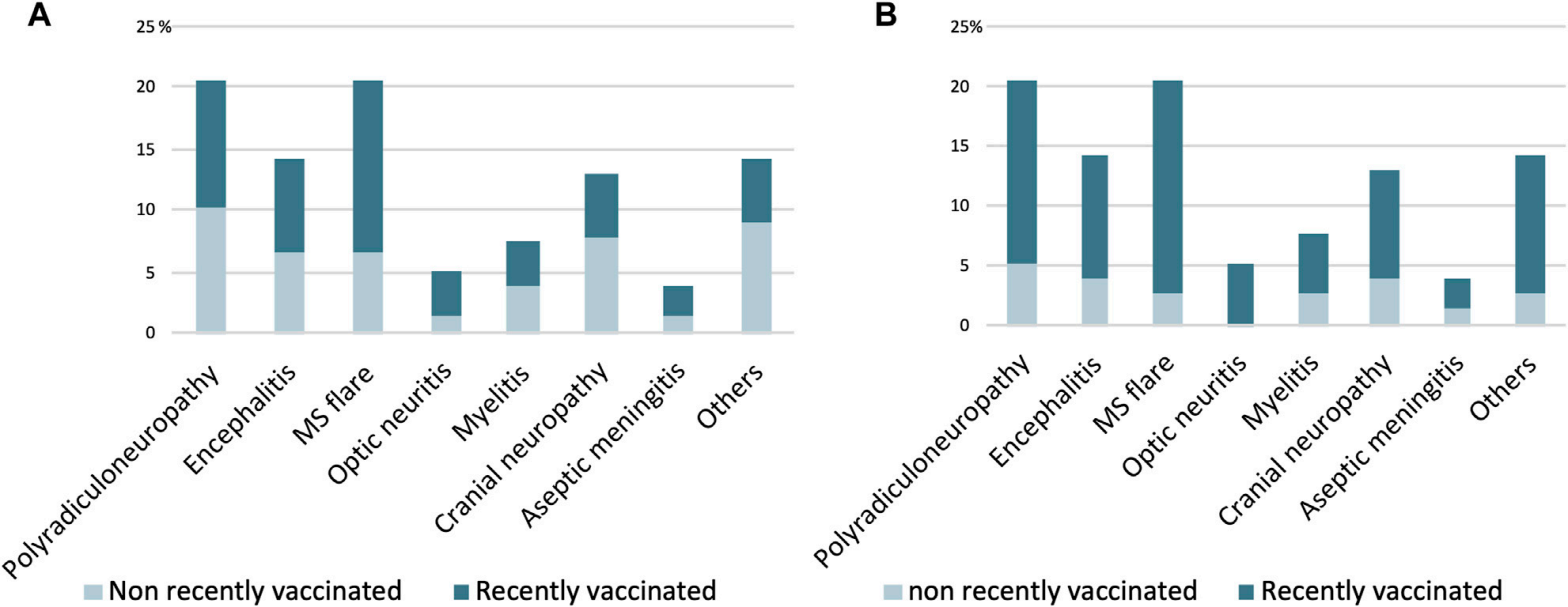
Neurological syndromes of probable autoimmune etiology in COVID19 vaccinated and non-vaccinated patients at 90 **(A)** and 30 **(B)** days prior. The Y axis represents the percentage of patients.

At V90 group, the most frequent disorders were polyradiculoneuropathies (22%), cranial neuropathies (16%), multiple sclerosis flares and encephalitis (both 14%). At NV90, multiple sclerosis relapses (27%), polyradiculoneuropathies (19%), and encephalitis (15%) (Figure 4).

**FIGURE 5:**
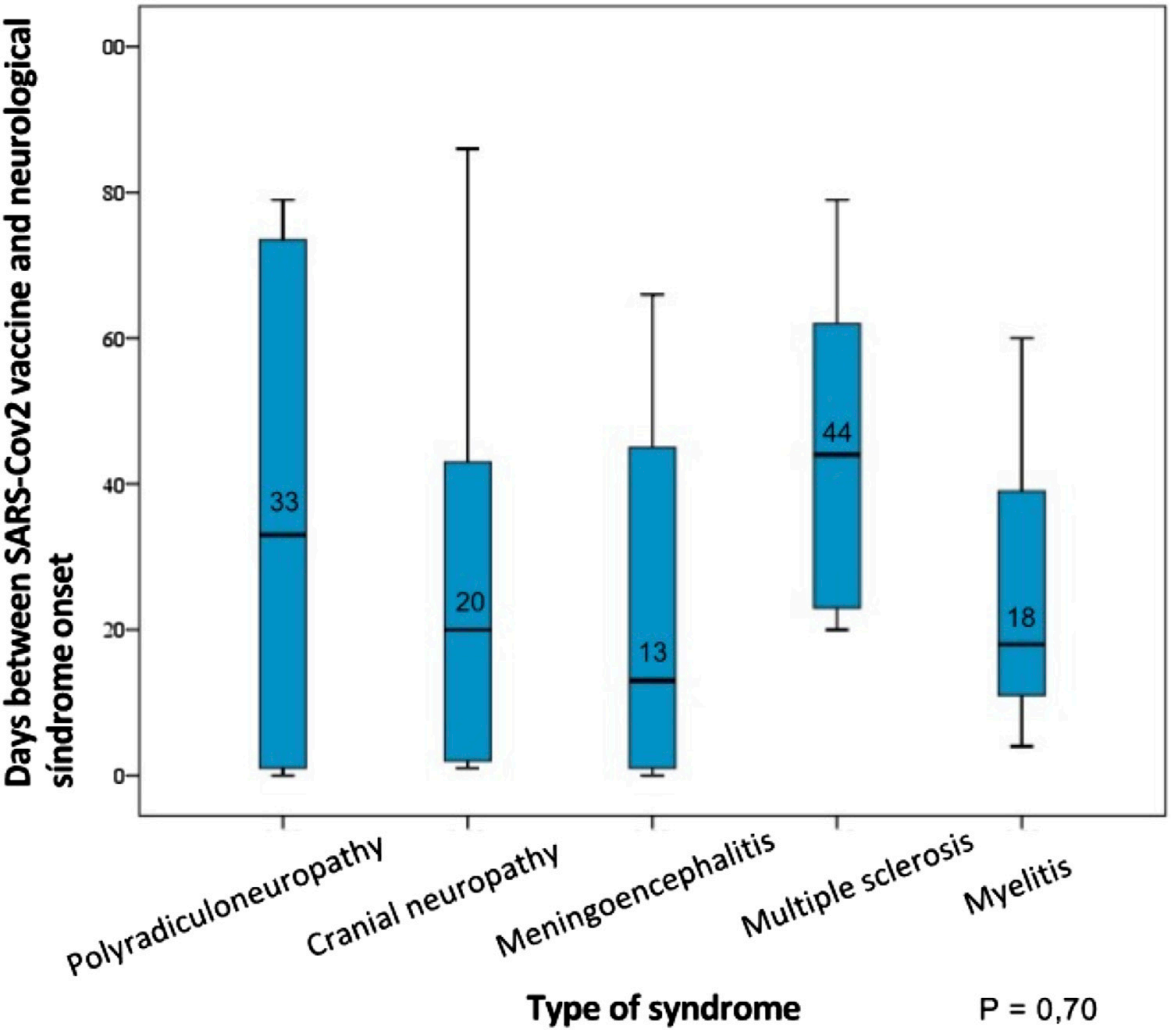
Time between SARS-Cov2 vaccination (days) and the onset of neuroimmune symptoms according to the type of syndrome presented.

The median number of days between vaccination and onset of neuroimmune symptoms was 33 days (SD 30.8) in the total sample: a) 44 days (SD 25.3) in multiple sclerosis relapses; b) 13 days (SD 27.7) in meningoencephalitis; c) 33 days in polyradiculoneuropathies (SD 34.0); d) 20 days in cranial neuropathies; and e) 18 days (SD 29.1) in myelitis. No significant differences were found comparing these times (*p* = 0.70) (Figure 5).

### 3.5 Neurological disorders depending on type of SARS-Cov2 vaccine

The most frequent syndromes between those that recently received the *BioNTech/Pfizer* vaccine (V90 n = 24) were polyradiculoneuropathies (n = 5), cranial neuropathies (n = 4) and encephalitis (n = 3).

Regarding *Oxford/AstraZeneca* group (V90 n = 5), the most frequent were polyradiculoneuropathies (n = 2), encephalitis (n = 1) and cranial neuropathies (n = 1).

**FIGURE 6:**
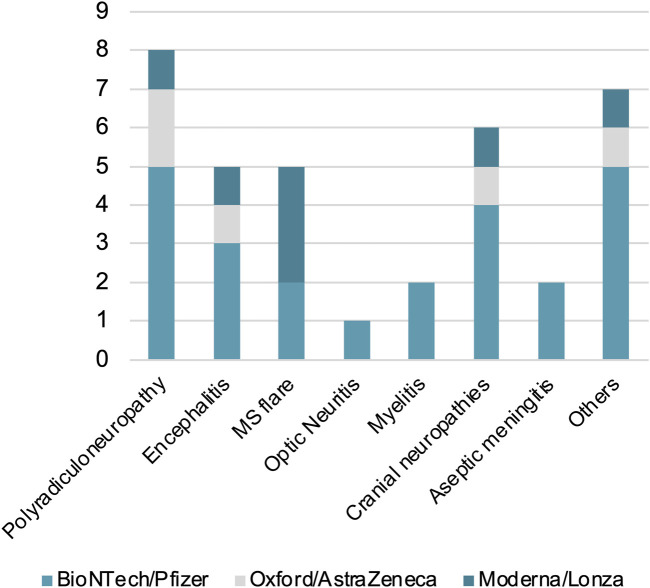
Dysimmune neurological disorders in patients recently vaccinated (V90) with BioNTech/Pfizer, Oxford/AstraZeneca or Moderna/Lonza vaccines. The Y axis represents the number of patients.

Concerning *Moderna/Lonza* vaccine (V90 n = 7), multiple sclerosis relapses (n = 3), polyradiculoneuropathies (n = 1), encephalitis (n = 1) and cranial neuropathies (n = 1) were the most frequent (Figure 6).

### 3.6 Post-vaccinated neurological syndromes features

In V90 group, 3/6 of cranial neuropathies were VII nerve palsies, 2/6 cases cranial multineuritis and 1/6 case III nerve palsy.

The majority of polyradiculoneuropathies (PNP) in the V90 group were demyelinating type (63%) and had mixed sensory-motor impairment (63%).

In this sample, most cases had suffered from their first multiple sclerosis relapse after vaccination, as only one V90 (2.78%) had a prior diagnosis of recurrent-remittent MS (McDonald MS criteria, 2017). There were none with a prior diagnosis of clinically isolated syndrome. The average number of lesions (old and new) in the MRI was 15 (SD 29.3). The juxtacortical, periventricular and infratentorial regions were affected in all cases; and only one case didn’t have spinal cord lesions.

In V90, during the inpatient study, one of three cases of myelitis (33%) was antiaquaporine-4 (AQ4) antibody positive and had a longitudinally extensive lesion on MRI, with a suspected diagnosis of Neuromyelitis optica spectrum disorder; another case had positive ANA antibodies (and no diagnostic criteria of systemic autoimmune disorders).

Only one of patients with encephalitis had visible lesions in magnetic resonance imaging, while the rest had a diagnosis based on clinical and cerebrospinal fluid findings.

Regarding optic neuritis in the V90 group, the only case had no spinal cord lesions neither optic nerve hyperintensities in magnetic resonance imaging and studied antibodies (anti-AQ4 and anti-MOG) were negative.

Except for the above-mentioned case with a prior diagnosis of multiple sclerosis, none of the other patients had been previously diagnosed with, or had exhibited symptoms indicative of, a neuroimmune syndrome.

### 3.7 Treatment and clinical evolution

61.1% of V90 and 66.6% NV90 patients received some type of immunomodulatory treatment (V90 n = 22; NV90 n = 28), that included intravenous corticosteroid therapy (V90 n = 10; NV90 n = 14), oral corticosteroids (V90 n = 6; NV90 n = 5), plasmapheresis (V90 n = 5; NV90 n = 5) and intravenous immunoglobulins (IV Igs) (V90 n = 1; NV90 n = 4).

**FIGURE 7:**
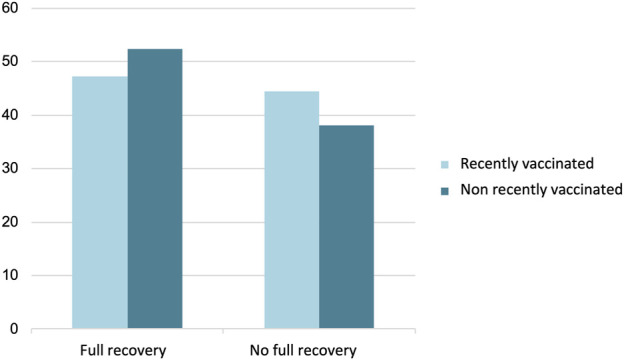
Clinical evolution 1 year after presenting neurological symptoms (n = 71). The Y axis represents the percentage of patients.

One year after presenting with neurological symptoms, 17 cases (47.2%) of those vaccinated and 22 cases (52.4%) of those not vaccinated in the previous 90 days had completely improved their neurological symptoms (Figure 7).

### 3.8 Clinical pharmacology assessment: causality algorithm and lymphocyte transformation test

Following a thorough neurological evaluation that confirmed a diagnosis and raised suspicion of a vaccine-related cause, 11 patients were referred to Clinical Pharmacology department for further assessment. The initial SPVS algorithm identified a conditional relationship (1–3 points) in 3 patients, a possible relationship (4–5 points) in 4 patients and a probable relationship (6–7 points) in 4 patients.

To further investigate potential immune responses to specific vaccine components in patients identified by the algorithm LTT was additionally performed. Four patients showed positive responses to polyethylene glycol 2000 and polysorbate 80 on LTT. Neurological presentations varied: One patient suffered from a III and V cranial neuropathy, another developed a facial multineuritis, and the remaining two patients presented with acute inflammatory demyelinating polyradiculoneuropathy. The final SPVS score identified a possible relationship (4–5 points) in six patients and a probable relationship (6–7 points) in five patients. Table 2 summarizes the type and latency of adverse reactions, along with the corresponding final algorithm scores and LTT results.

**TABLE 2:** Type and latency of adverse reactions, along with the corresponding algorithm scores and LTT results.

Patient	Vaccine	Adverse reaction	Latency (Days)	SPVS algorithm (Score)	LTT result (SI)
1^a^	BNT162b2 (Pfizer-BioNTech)	Acute inflammatory demyelinating polyradiculoneuropathy	36	6	Negative (1.4)
2^a^	BNT162b2 (Pfizer-BioNTech)	Acute inflammatory demyelinating polyradiculoneuropathy	2	4	Negative (1.6)
3	BNT162b2 (Pfizer-BioNTech)	Acute inflammatory demyelinating polyradiculoneuropathy	1	4	Positive (3.8)
4^a^	mRNA-1273 (Moderna)	Acute Transient Encephalopathy	1	4	Negative (1.1)
5	BNT162b2 (Pfizer-BioNTech)	Optic neuritis	35	4	negative (1.3)
6	BNT162b2 (Pfizer-BioNTech)	Non convulsive status epilepticus	2	4	Negative (1.0)
7^a^	ChAdOx1-S (AstraZeneca)	Cranial nerves III and IV neuropathy	2	6	Positive (4.6)
8	BNT162b2 (Pfizer-BioNTech)	Facial multineuritis, erythema multiforme	33	6	Positive (4.9)
9^a^	mRNA-1273 (Moderna)	Encephalomyelitis, left hypoglossal nerve paresis	6	6	Negative (0.5)
10^a^	BNT162b2 (Pfizer-BioNTech)	Acute inflammatory demyelinating polyradiculoneuropathy	3	4	Positive (10.7)
11^a^	BNT162b2 (Pfizer-BioNTech)	Aseptic meningitis	1	6	Negative (0.4)

^a^ Cases previously reported in the literature (Cabanillas and Novak, 2021). SI, Stimulation index; SPVS, Spanish pharmacovigilance system.

## 4 Discussion

Many severe neurological manifestations have been described following exposure to COVID-19 vaccine. The four basic principles proposed by the WHO that suggest an association between vaccination and adverse event included: a) consistency and strength (similar results should be obtained by different studies), b) specificity (the adverse event should be linked specifically to the vaccine) and c) temporal relationship (vaccination must precede the event) (Wraith et al., 2003). Such strict criteria, especially the specificity one, are challenging to meet in vaccine-triggered immunomediated syndromes, as they can also occur spontaneously without a clear trigger or induced by other factors. This is the reason why we have tried to identify clinical peculiarities of these syndromes in vaccinated patients.

This study describes a series of cases of severe neuroimmune disorders, which required admission to the Neurology Department of the Hospital La Paz, in temporary association with vaccination against SARS-CoV-2 in 90 days prior. The median time between vaccination and clinical onset was 33 days, compared to previous reviews and case studies, that described a median of 9 and 11 days (Ismail and Salama, 2022; Kaulen et al., 2022). The risk decreases over time, moreover, a previous study found the greatest risk of Guillain-Barré syndrome 29–35 days after *Oxford/AstraZeneca vaccination* (IRR, 1.55; 95% CI: 1.03–2.34) (Patone et al., 2021), that is similar to our study.

The most frequent neuroimmune syndromes were polyradiculoneuropathies, multiple sclerosis relapses and cranial neuropathies. This is partly consistent with previous studies, such as a review by Garg and Paliwal (2022), who describe acute inflammatory demyelinating polyradiculoneuropathy (AIDP), followed by cranial nerve involvement (mainly facial) as the most frequent neurological events. Another study from 2021, by Finsterer, (2022) describes acute inflammatory demyelinating polyradiculoneuropathy as the most frequently encountered disorder after headaches (most of these being moderate or mild), venous sinus thrombosis, transverse myelitis and facial palsy. There is a concern that vaccination, by inducing certain immune responses, could trigger a flare-up in patients with a prior immune-mediated neurological syndrome, including multiple sclerosis (Jeon et al., 2023). A high frequency of multiple sclerosis first relapses has been seen in our sample, in both vaccinated and non-vaccinated groups. However, the findings of the prospective observational study by Blanco et al., (2023) suggest that mRNA COVID-19 vaccination is safe and does not exacerbate symptoms in multiple sclerosis patients. In our study, we were unable to estimate the relapse frequency in the general multiple sclerosis population because, at our hospital, they are typically treated as outpatients in a day hospital, and were thus not included.

Treatment included administration of corticoids, plasma exchange and IV Igs, an overall good response has been seen, similar to previous studies. Kaulen et al., (2022) found complete and partial remision of the symptoms in 71% of patients, 5% had stable disease and none progressed.

The most frequently administered vaccines in patients suffering from a neurological disorder in our sample were *Comirnaty* by *Pfizer-BioNTech* (63.9%), followed by *Spikevax* by *Moderna* (19.4%) and *Vaxzevria* by *AstraZeneca-Oxford* (13.9%), probably reflecting the frequency of vaccination with these subtypes in this population. Since the beginning of the vaccination campaign in Spain (on 27 December 2020) until 31 December 2022, 111,293,866 vaccine doses had been administered: 60.4% original *Comirnaty*, 21.7% original *Spikevax*, 8.8% *Vaxzevria,* 7.1% bivalent (original/omicron) *Comirnaty*, 1.8% *Jcovden by Janssen* and 0.2% bivalent *Spikevax* (aemps, 2024). The first two use genetically engineered RNA, while *AstraZeneca’*s one uses viral vectors. The other types of vaccines: inactivated virus (*Sinovac*) and protein subunits vaccines (*Novavax*) were not approved in Spain by that time. Even though there are no direct comparisons, no type of COVID-19 vaccine has shown a higher risk than others for immune-related neurological adverse reactions (Alonso Castillo and Martínez Castrillo, 2022), except for rare cases of cerebral venous sinus thrombosis related to thrombosis with thrombocytopenia syndrome after *Janssen*’s vaccine (Mascellino et al., 2021).

Prior SARS-CoV-2 infection was taken into account, noticing that in vaccinated cases, the time between the infection and the onset of neuroimmune symptoms is higher than in non-vaccinated cases with prior SARS-CoV-2 infection (119 versus 12.5 days; Mann-Whitney’s U *p* = 0.014). The higher temporal association of neurological syndromes and COVID-19 in the non-recently vaccinated patients could be explained by the postinfectious immune trigger in some patients of this subgroup (Carod-Artal, 2020; Harapan and Yoo, 2021). Although the temporal co-existence of vaccination and infection could enhance the risk of immune disorders, we haven’t been able to demonstrate it. Nor have we observed a high proportion of a history of autoimmune diseases in the patients in our study.

Overall, the incidence of neuroimmune syndromes in temporal association with the SARS-CoV-2 vaccine that require hospitalization is low compared to the incidence of severe SARS-CoV-2 disease without vaccination. In addition, the outcome of these diseases is mostly favorable. The benefits of vaccinations outweigh the comparatively small risks. However, as annual vaccination campaigns will be necessary because of virus outbreaks that persist to this day, and causality assessment by algorithms have low specificity and negative predictive values (Macedo et al., 2006), it is necessary to develop new biomarkers that can be applied in clinical practice.

SARS-CoV-2 vaccine antigens may trigger autoimmune responses by molecular mimicry, bystander activation or epitope spreading. Molecular mimicry takes place because self-antigens share a sequence or structural similarities with vaccine antigens. In bystander activation, the vaccine activates antigen presenting cells that, in turn, could activate autoreactive T cells or cause the release of cytokines that could harm neighboring cells. On the other hand, in epitope spreading, antigenic epitopes non-cross-reactive with an inducing epitope become additional targets of an immune response. These pathways result in synthesis and release of cytokines like interleukin (IL)-1, IL-6, tumor necrosis factor-alpha, and prostaglandin-E2 into the bloodstream, activating a proinflammatory cascade that can affect other body systems and ultimately cause neuroinflammation (Patone et al., 2021; Zlotnik et al., 2021; Kaulen et al., 2022).

Adjuvants could play an important role in the generation of these neuroimmune syndromes. They are substances added to a vaccine to stimulate the magnitude and increase the duration of the immune response. There are several different compounds used to this date. Some of them are oil-based adjuvants, virosomes, toll-like receptors related adjuvants, unmethylated CpG dinucleotides or tuftsin (Guimarães et al., 2015). The adjuvants used in COVID-19 vaccines are polyethylene glycol 2000 in *BioNTech/Pfizer* and *Moderna* vaccines, and polysorbate 80 in *Janssen* and *AstraZeneca* vaccines. The latter had been previously used in other vaccines such as human papilloma virus or influenza vaccine, with several neuroimmune syndromes being reported, including autoimmune syndrome induced by adjuvants (ASIA) syndrome, transverse myelitis or Guillain-Barre syndrome (Juurlink et al., 2006; Vadalà et al., 2017).

In our study, PEG2000 and P80 have been investigated as possible triggers of immunity stimulation by using the LTT. As a pilot study, the sample size is small. However, in a recent study, LTT has been positive in 50% of neurological reactions after COVID-19 vaccine (Ruiz-Fernández et al., 2023). This reflects a possible delayed hypersensitivity to excipients in these patients that should be assessed in order to make recommendations of future vaccine doses administration. In our centre, LTT is implemented in clinical practice as part of the study of vaccines and other drugs adverse reactions. If LTT is positive, the specific drug is contraindicated, although patients are reassessed every 6 months, because adjuvants used in these cases are alcohols and the sensitization may disappear (Ruiz-Fernández et al., 2023).

This case study has some limitations as its retrospective condition, the small size of the sample and a low proportion of patients referred to Clinical Pharmacology for assessment and TTL study. Furthermore, only hospitalized patients were included in the study. Individuals with mild symptoms might have not consulted our institution and from those who consulted the emergency department, only patients with severe syndromes that required monitoring and further investigation were hospitalized. In addition, cases of cerebrovascular disease, such as venous sinus thrombosis, vaccine-induced immune thrombotic thrombocytopenia, or intracranial hemorrhage were not included.

In conclusion, few differential clinical characteristics have been seen in patients with neuroimmune disorders after COVID-19 vaccination, which must be confirmed in larger studies. A favorable clinical course is the norm. In addition to causality algorithms, LTT can be a useful tool to know pathophysiological mechanisms of neurological manifestations after vaccination and make recommendations about the introduction of new vaccine doses.

## Data Availability

The raw data supporting the conclusions of this article will be made available by the authors, without undue reservation.
